# Defect‐Cascades‐Induced Photodegradation in InP/ZnSe/ZnS Quantum Dots

**DOI:** 10.1002/advs.202515691

**Published:** 2025-11-03

**Authors:** Yeongrok Jin, Seongmun Kim, Yeo‐Geon Yoon, Taekjoon Lee, Jae Bok Chang, Kyung Sig Lee, Nari Ahn, Ran Kim, Young‐gil Park, Jaekwang Lee

**Affiliations:** ^1^ Department of Physics Pusan National University Busan 46241 Republic of Korea; ^2^ Institute of Industrial Science The University of Tokyo Tokyo 153–8505 Japan; ^3^ Samsung Display Co., Ltd. Giheung‐gu Yongin‐si Gyeonggi‐do 17113 Republic of Korea

**Keywords:** DFT calculations, electron spin resonances, quantum dots, sulfur vacancies, type‐II band alignments, vacancy pairs

## Abstract

By integrating light‐induced in situ electron spin resonance (ESR) spectroscopy with density functional theory (DFT) calculations, it is revealed that surface sulfur vacancies (s‐V_S_) in InP/ZnSe/ZnS quantum dots (QDs) act as critical initiation centers for cascade defect formation, directly compromising photostability and optoelectronic performance of QDs. Blue‐light irradiation initiates defect propagation from s‐V_S_, inducing sequential formation of Zn, S, and Se vacancies that extend toward the ZnSe/ZnS interface. This defect cascade disrupts the entire band alignment, shifting it from type‐I to type‐II and drastically reducing external quantum efficiency (EQE). By contrast, the targeted passivation of s‐V_S_ sites effectively suppresses defect evolution, thereby enhancing photostability and preserving high EQE under prolonged illumination. These findings establish s‐V_S_ as a principal accelerator of light‐induced degradation, underscoring the necessity of surface defect control for developing highly stable and high‐performance QDs.

## Graphical abstract

Surface sulfur vacancies in InP/ZnSe/ZnS quantum dots trigger vacancy cascades under blue light, propagating toward the ZnSe interface. In situ ESR (*g* = 2.004, 2.012) and DFT reveal a defect‐driven shift from type‐I to type‐II band alignment, reducing radiative recombination and EQE. Passivating s‐V_S_ suppresses cascades, preserving photostability and device performance.

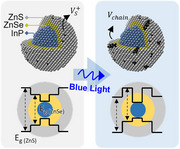

## 1 Introduction

Colloidal quantum dots (QDs) offer tunable emission wavelengths through size‐dependent quantum confinement effects and exhibit excellent optical properties, including high emission efficiency and narrow emission linewidths.^[^ ^1^, ^2^, ^3^ ^]^ These attributes have propelled QDs into various advanced applications, notably in lighting, displays, and bioimaging.^[^ ^4^, ^5^, ^6^ ^]^ Among groups II–IV chalcogenide QDs, such as CdS^[^ ^7^, ^8^ ^]^ and CdSe,^[^ ^9^, ^10^, ^11^ ^]^ their high luminescence has driven commercial deployment in displays. However, the toxicity and environmental risks associated with cadmium‐ and lead‐based QDs considerably restrict their practical applications.^[^ ^12^ ^]^

By contrast, indium phosphide (InP) QDs have emerged as leading nontoxic alternatives.^[^ ^13^, ^14^ ^]^ InP QDs achieve tunable visible emission via size control and offer high color purity, declaring them as promising candidates for optoelectronic technologies. However, InP QDs often exhibit lower photoluminescence (PL) quantum yields and inferior photostability than their CdSe counterparts, primarily owing to chemical instability.^[^ ^15^ ^]^ High oxygen susceptibility of InP often leads to rapid surface oxidation, which facilitates the formation of a high density of surface trap states that undermine emission efficiency and broaden spectral linewidths. In particular, exposure to air, especially under moisture and oxygen environments, substantially compromises the optical and chemical stability of InP QDs and thus limits their practical applications.^[^ ^16^, ^17^, ^18^, ^19^ ^]^ To address these limitations, core/shell structural strategies have been widely adopted. Encapsulating the InP core with wide‐bandgap semiconductors, such as ZnS and ZnSe, enhances luminescence efficiency and chemical stability. A dual‐shell structure—in which InP core is first overcoated with ZnSe inner shell and then ZnS outer shell—has been developed to exhibit high PL quantum yields in InP QDs. The outer shell restricts excitons in the core while protecting it from the external environment, whereas the inner shell relieves the lattice mismatch between InP and ZnS and suppresses the formation of defects at the core–shell interface.^[^ ^20^, ^21^, ^22^ ^]^ However, even with these advances, the underlying mechanisms of photodegradation in InP/ZnSe/ZnS QDs, i.e., the gradual decline in emission intensity when exposed to continuous blue light—remain poorly understood.^[^ ^23^, ^24^, ^25^ ^]^

A key challenge is the lack of direct and real‐time observation of defect formation and evolution during device operation. Conventional optical and structural analyses provide only limited insights into the dynamic behavior of these photoactivated defects during continuous illumination. By contrast, ESR spectroscopy is a powerful tool that offers high sensitivity to paramagnetic defects, including lattice vacancies and surface radicals that possess unpaired electron spins, enabling fingerprint‐like identification of defect species through their unique *g*‐values.^[^ ^26^ ^]^ In general, ESR facilitates the quantitative analysis of their concentrations and real‐time tracking of their evolution under illumination. Despite its advantages, ESR has been mainly applied to bulk and colloidal II–VI semiconductors, such as ZnS, ZnSe, and ZnO.^[^ ^26^, ^27^, ^28^, ^29^, ^30^ ^]^ Few studies have explored in situ ESR for investigating real‐time defect evolution during optical excitation in InP/ZnSe/ZnS QDs.

Herein, we integrate in situ ESR spectroscopy with DFT calculations to elucidate the real‐time evaluation of defect states in InP/ZnSe/ZnS core/double‐shell QDs under blue‐light irradiation. This approach enables a detailed understanding of the photodegradation mechanism and evaluation of its influence on the optoelectronic structure and photophysical properties of QDs at the atomic scale. We identify surface S vacancies (s‐V_S_) on the ZnS outer shell as the earliest and most prominent ESR signature (*g* = 2.004), serving as nucleation centers for subsequent defect cascades. Prolonged illumination results in the sequential appearance of additional ESR signatures at *g* = 2.012 and *g* = 1.957, attributed to the zinc vacancies (V_Zn_) and oxygen‐related defect states, respectively, indicating a cascading defect formation accompanied by the structural and electronic transformation at the core/shell interface. These evolving defect populations directly correlate with a transition in band alignment from type‐I (carrier confinement in the InP core) to type‐II (spatial separation of electrons and holes), resulting in reduced PL intensity and external quantum efficiency. By contrast, QDs with lower s‐V_S_ concentrations exhibited stable ESR spectra and retained high PL intensity, confirming the key role of s‐V_S_ in determining the photodegradation of InP‐based QDs.

## 2 Results and Discussion

### 2.1 ESR Analysis of QDs with and Without Initial s‐V_S_ Vacancies

**Figure 1:**
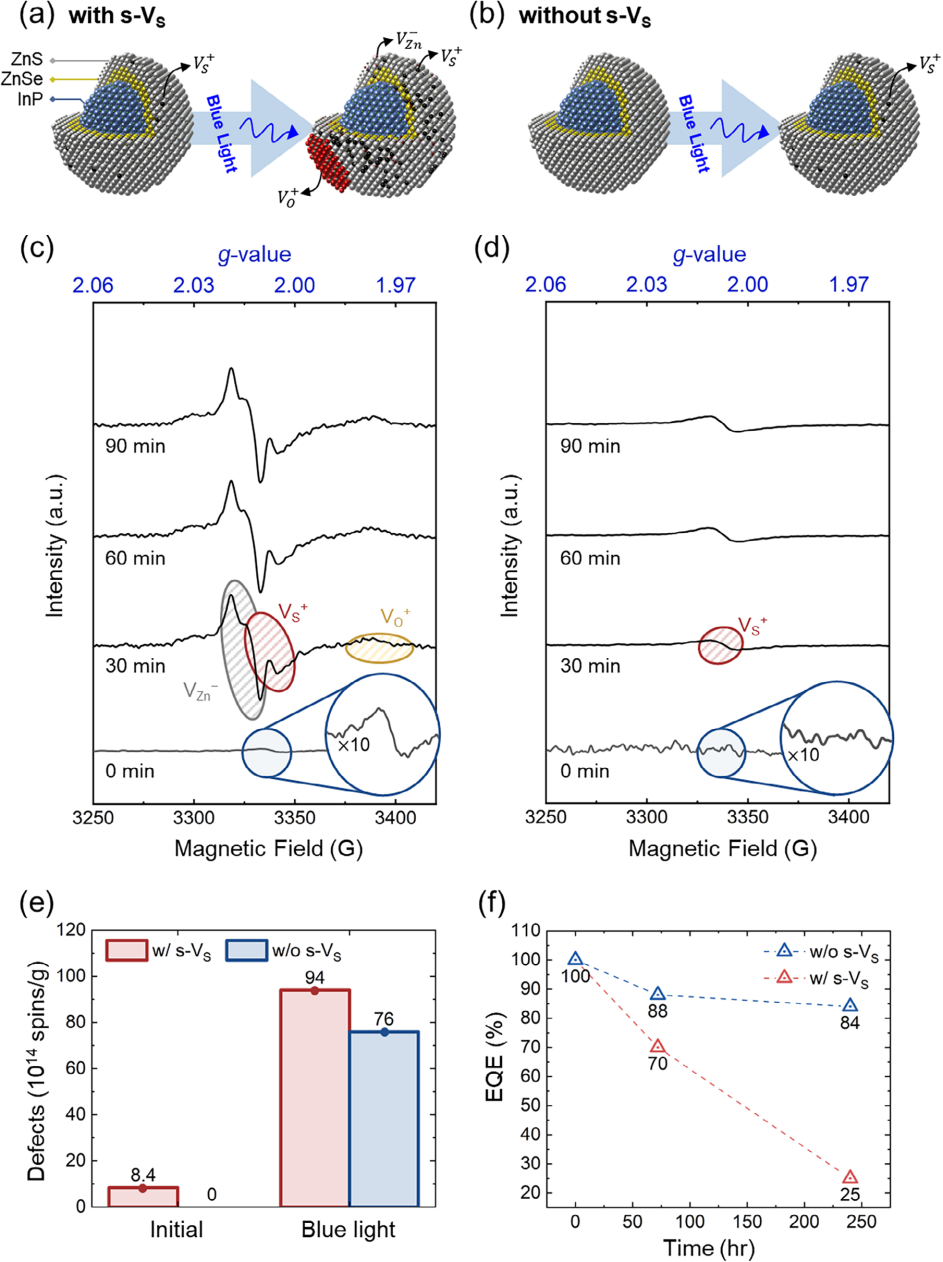
ESR experimental results for InP/ZnSe/ZnS QDs with and without initial S vacancies. (a) and (b) are schematic representations of the photodegradation process in QDs: (a) with initial S vacancies and (b) without initial S vacancies. In the QD structure, the blue core represents InP, the yellow inner shell is ZnSe, the gray outer shell is ZnS, the red indicates oxygen, and black dots represent vacancies. (c) and (d) show the corresponding ESR spectra for QDs (c) with and (d) without initial s‐V_S_, recorded at 0, 30, 60, and 90 min (the 0‐min signal is magnified by 10x). Peaks corresponding to different defect types are labeled in the spectrum. e) Quantification of defects in QDs as detected in ESR spectroscopy. f) EQE comparison between enhanced and conventional QDs.

Time‐resolved ESR measurements were performed on QDs with and without pre‐existing s‐V_S_ under blue‐light irradiation. **Figure** **1**a schematically illustrates the structural evolution of QDs with pre‐existing s‐V_S_, whereas Figure 1b depicts the scenario for QDs without such initial defects. In the QDs with pre‐existing s‐V_S_ (refer to Figure 1c), the ESR spectrum at 0 min exhibited a weak signal at 3338.4 G (*g* = 2.004), which is characteristic of V_S_. To determine the origin of this peak, additional ESR simulations for individual materials will be discussed in the next section. As the blue‐light irradiation progressed, spectra recorded at 30, 60, and 90 min exhibited drastic changes, indicating the dynamic evolution of defect states. Peak decomposition, performed using the derivative of Gaussian functions, revealed three distinct signals: two sharp peaks at 2.012 (V_Zn_) and 2.004 (V_S_) and a broad peak at 1.957 (O‐related defects).

The *g* = 2.012 signal corresponds to negatively charged Zn vacancies (V_Zn_^−^), consistent with previous reports on nanostructured ZnS.^[^ ^31^ ^]^ Meanwhile, the broad signal at *g* = 1.957 is owing to O‐related defects in ZnO, most likely originating from unpaired electrons in O vacancies, which act as donors.^[^ ^32^, ^33^, ^34^, ^35^ ^]^ The broad nature of this peak indicates a relatively low O vacancy concentration and inhomogeneous surface oxidation, with signal broadening caused by variations in spin orientation, coordination environments, and oxidation states. These results reveal that blue‐light irradiation induces a cascade of defect formation, starting with s‐V_S_ and propagating to V_Zn_ and surface oxidation.

In QDs lacking initial s‐V_S_, no detectable ESR peaks were observed at 0 min (Figure 1d). After blue‐light irradiation, a weak peak at *g* = 2.004 emerged, thereby confirming the formation of s‐V_S_ during irradiation. However, the absence of pre‐existing s‐V_S_ substantially delayed the onset and propagation of further defects, suggesting a catalytic role of s‐V_S_ in initiating defect formation.

Figure 1e compares the defect densities for QDs with and without the initial s‐V_S_ after controlled blue‐light irradiation. The data revealed that QDs with pre‐existing s‐V_S_ exhibited a substantially higher defect density than their vacancy‐free counterparts (for longer‐time data, see Figure S4, Supporting Information). To connect material‐level defects to device performance, Figure 1f presents the corresponding external quantum efficiency (EQE) evolution under prolonged stress: the vacancy‐free devices stabilize at high efficiency, while devices with s‐V_S_ continue to degrade, underscoring the adverse impact of s‐V_S_ on long‐term stability.

### 2.2 DFT‐Based ESR Spectrum Simulation in ZnS/ZnSe‐Shelled QDs

**Figure 2:**
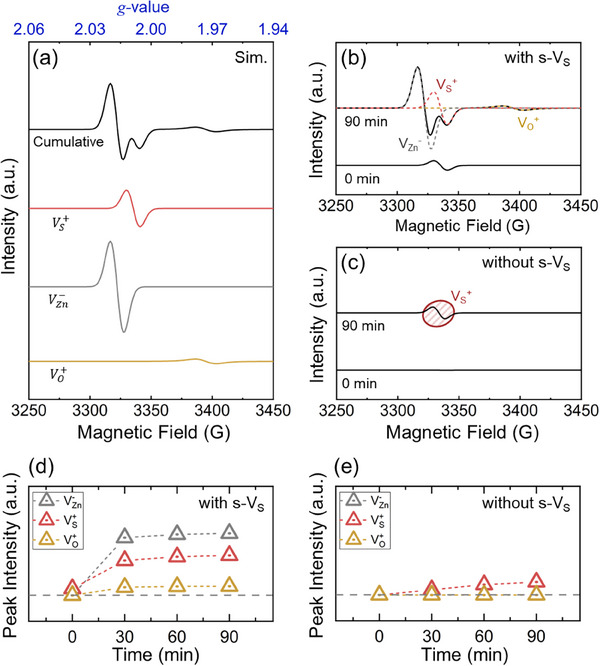
ESR spectrum simulation for individual defect contributions. a) The black solid line represents the cumulative intensity, while the colored lines show the individual spectra of each defect type. b,c) Cumulative ESR spectra at 0 and 90 min (b) with and (c) without initial surface sulfur vacancies (s‐V_S_). d,e) Time‐dependent changes in the signal intensities for V_Zn_^−^, V_S_^+^, and V_O_^+^ defects during irradiation, displayed for the cases (d) with and (e) without initial s‐V_S_.

To further understand the g‐value shifts associated with specific defect types, we performed DFT calculations on intrinsic point defects in ZnO, ZnS, and ZnSe using the Gauge Including Projector Augmented Waves (GIPAW) method. Isotropic g‐values for Zn and X (S, Se, and O) vacancies were computed, focusing on random QD orientations. **Table** **1** summarizes the calculated g‐values, efficiently aligning with experimental observations. ESR spectrum simulations for each defect type were conducted at 9.36 GHz to replicate the experimental setup. The cumulative spectrum, shown in **Figure** **2**a, combines the individual contributions from s‐V_S_, V_Zn_^−^, and O‐related defects. This simulated spectrum closely reproduces the experimental ESR results for QDs with pre‐existing s‐V_S_, thereby confirming the accuracy of the DFT‐based approach.

**Table 1:** Calculated g‐values.

Defect type	ZnO	ZnS	ZnSe
Zn vacancy	2.009	2.013	2.039
*X* vacancy	1.970	2.005	2.006
Zn interstitial	2.0025	1.996	2.000
*X* interstitial	2.006	2.001	1.714

Figure 2b presents the time‐dependent ESR spectra for the QDs with pre‐existing s‐V_S_. At 0 min, only a weak S vacancy signal is observed. After 90 min of blue‐light irradiation, distinct peaks corresponding to V_Zn_^−^, V_S_, and O‐related defects become apparent. Notably, the dominant peak at g = 2.004 confirms the prevalence of V_S_, even as charge compensation mechanisms lead to the formation of V_Zn_^−^. Interestingly, ZnSe‐related defects (V_Se_) were not detected, likely because ZnSe is encapsulated within ZnS in the shell structure, with the surface s‐V_S_ having the dominant role in defect formation under blue‐light irradiation. This finding highlights the critical influence of shell surface composition on defect evolution.

These results are consistent with earlier theoretical studies. For ZnS, previous research demonstrated that V_S_ and Zn interstitials are dominant under p‐type conditions, whereas V_Zn_ is more prevalent in n‐type environments, consistent with our experimental observations.^[^ ^36^ ^]^ A singly negatively charged Zn vacancy (V_Zn_^−^) introduces an unpaired electron localized within the p orbital of a neighboring S atom, resulting in the characteristic *g*‐value shifts observed in the ESR spectra. The observed g‐shift primarily arises from the coupling between the spin and orbital angular momentum of the unpaired electron. According to perturbation theory, when the dominant contribution to the g‐shift originates from orbitals localized at a specific atom via spin–orbit coupling, the g‐tensor can be expressed as follows:

(1) $$g_{ij}=g_{e}\;\delta_{ij}+2\lambda \Lambda_{ij}$$

where *δ_ij_* is a Kronecker delta, *λ* is the spin orbit coupling (SOC) constant of the dominant atom, and the matrix elements Λ_*ij*_ are defined as follows:

(2) $$\Lambda_{ij}=\sum_{n\neq 0}\frac{\langle 0|{\hat{l}}_{i}|n\rangle \langle n|{\hat{l}}_{j}|0\rangle }{\in_{0}-\in_{n}}\;$$

here, *i* and *j* index the Cartesian directions *x*, *y*, and *z*; the operators ${\hat{l}}_{x},{\hat{l}}_{y},$ and ${\hat{l}}_{z}$ are the components of the angular momentum components; |*n*〉 denotes nearby empty or filled states virtually accessed via SOC; and *ϵ_n_* are their corresponding energies. Within the standard second‐order perturbative framework, the g‐shift scales inversely with the energy separations | ∈ _0_ − ∈ _*n*_| and originates from spin–orbit–weighted angular‐momentum matrix elements between ∣0⟩ and nearby empty/filled states ∣n⟩. Hence, smaller denominators and larger SO constants (*λ*) enhance |Δ*g*|.

Figure S2 (Supporting Information) presents the projected DOS (pDOS) for the Zn*X* system (*X* = O, S, Se) with vacancies, highlighting defect levels near valence band maximum (VBM)/conduction band minimum (CBM). In Figure S2a,c,e (Supporting Information), the pDOS on *X* atoms adjacent to V_Zn_ shows a depletion of the down‐spin states and a shift of up‐spin states; the unpaired spin resides mainly on the nearest‐neighbor chalcogen *p* orbitals. Two cooperative trends then emerge across the O → S → Se. First, the relevant DOS separation decreases (VBM–*E_F_* = 1.56, 0.25, and ≈0 eV in ZnO, ZnS, and ZnSe, respectively; Figure S2, Supporting Information), which strengthens the second‐order coupling. Second, because the spin density has *X*‐*p* character, the effective SOC weighting increases with heavier chalcogens (*λ*_Se_ > *λ*_S_ > *λ*_O_). By contrast, for V_X_ (*X* = O, S, Se) the DOS shows occupied defect states just below *E*_F,_ and the relevant band‐edge spacings with respect to *E*_F_ do not evolve monotonically (CBM–*E*_F_ = 0.21, 0.96, 0.10 eV for ZnO, ZnS, ZnSe, respectively). Consequently, the *g*‐trend for V_X_ cannot be explained solely by simple band‐edge separation alone; additional effects are likely involved, and further investigations will be required. Regarding stability, V_Zn_ forms a sharp, highly localized acceptor state and readily stabilizes a negatively charged state, consistent with the DOS trends. In contrast, V_X_ is donor‐like and stabilizes a positively charged state.

### 2.3 Defect Cascades Initiated by s‐V_S_

**Figure 3:**
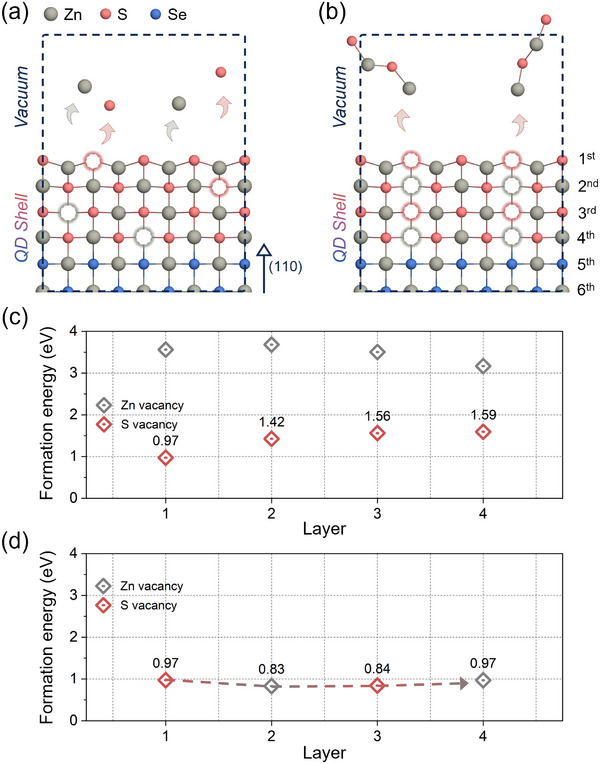
DFT slab calculations of vacancy formation energies in a ZnS/ZnSe heterostructure. a,b) Schematic illustrations of two vacancy formation pathways: independent formation (a) and sequential propagation (b). The (110) slab model consists of a ZnSe core (layers 5 and 6) and a ZnS shell (layers 1–4). Layers are numbered from the surface (layer 1) toward the bulk. Red open circles indicate S vacancies; gray open circles indicate Zn vacancies. c) Layer‐resolved formation energies of Zn and S vacancies under Zn‐rich conditions. d) Formation energies for sequential vacancy propagation, highlighting the reduced energetic barriers for both Zn and S vacancies under Zn‐rich conditions.

To deeply understand how surface defects propagate and affect the ZnS/ZnSe heterostructure, we constructed a ZnS/ZnSe slab exposing the (110) surface (**Figure** **3**). We acknowledge that actual QDs may expose multiple facets. In zinc blende ZnS, however, the (110) planes are nonpolar and exhibit the lowest surface energies, whereas (111) and (100) planes are polar and thus energetically less favorable. For this reason, the (110) surface is widely regarded as a standard and physically motivated choice for DFT calculations.^[^ ^37^, ^38^ ^]^ In addition, Wulff‐shape analyses identify {110} facets as the most abundant on ZnS nanocrystals.^[^ ^39^, ^40^ ^]^ The slab model enabled the layer‐by‐layer evaluation of the formation energies (*E*_form_) of Zn and S vacancies, with all calculations conducted under Zn‐rich conditions.

Figure 3a illustrates the slab structures with each vacancy site: V_Zn_ and V_S_. The vacancy positions were intentionally modeled at different depths to examine their behavior as a function of depth. Each atomic layer is labeled on the right side of the figure, with the 4th and 5th layers forming the interface region. Figure 3c presents the calculated formation energies across the layers. At the surface, the *E*_form_ of V_Zn_ and V_S_ were determined to be 3.56 and 0.97 eV, respectively, whereas in the bulk region, V_Zn_ showed a *E*_form_ of 3.17 eV, while V_S_ exhibited a higher value of 1.59 eV compared to that at the surface. This change highlights the preference for V_S_ to form at the surface rather than in the bulk. By contrast, V_Zn_ became energetically more favorable in deeper layers, especially near the ZnSe interface. This trend aligns with the rising VBM toward the ZnSe region, stabilizing the acceptor‐like V_Zn_. These results reveal that V_S_ is assumed to dominate at the ZnS surface, whereas V_Zn_ is more likely to emerge near the ZnSe interface.

To further explore the defect evolution, we examined sequential vacancy formation, beginning with s‐V_S_ and enabling the defects to propagate inward (Figure 3b). The sequential process initiates with the creation of s‐V_S_, followed by alternating V_Zn_ and V_S_ in the subsequent layers. This cascade of vacancies eventually reaches the ZnS/ZnSe interface, altering the local atomic structure and electronic properties. The calculated formation energies along the vacancy evolution are shown in Figure 3d. Notably, the formation energy of V_S_ considerably decreases as they propagate toward the bulk, indicating that sequential defect formation facilitates the stabilization of the deeper‐layer V_S_. This behavior contrasts with independent vacancy formation, where V_S_ formation energy increases with depth. The reduction in V_S_ formation energy during sequential propagation likely results from localized charge redistribution and stabilization effects from neighboring vacancies. The formation energy of V_Zn_ also decreases as the vacancy cascade progresses, driven by charge compensation effects. These results indicate that the initial V_S_ at the surface acts as a catalytic site, triggering a cascade of vacancy formation that extends through the ZnS/ZnSe interface.

### 2.4 Electronic Structure Changes by Sequential Vacancy Formation

**Figure 4:**
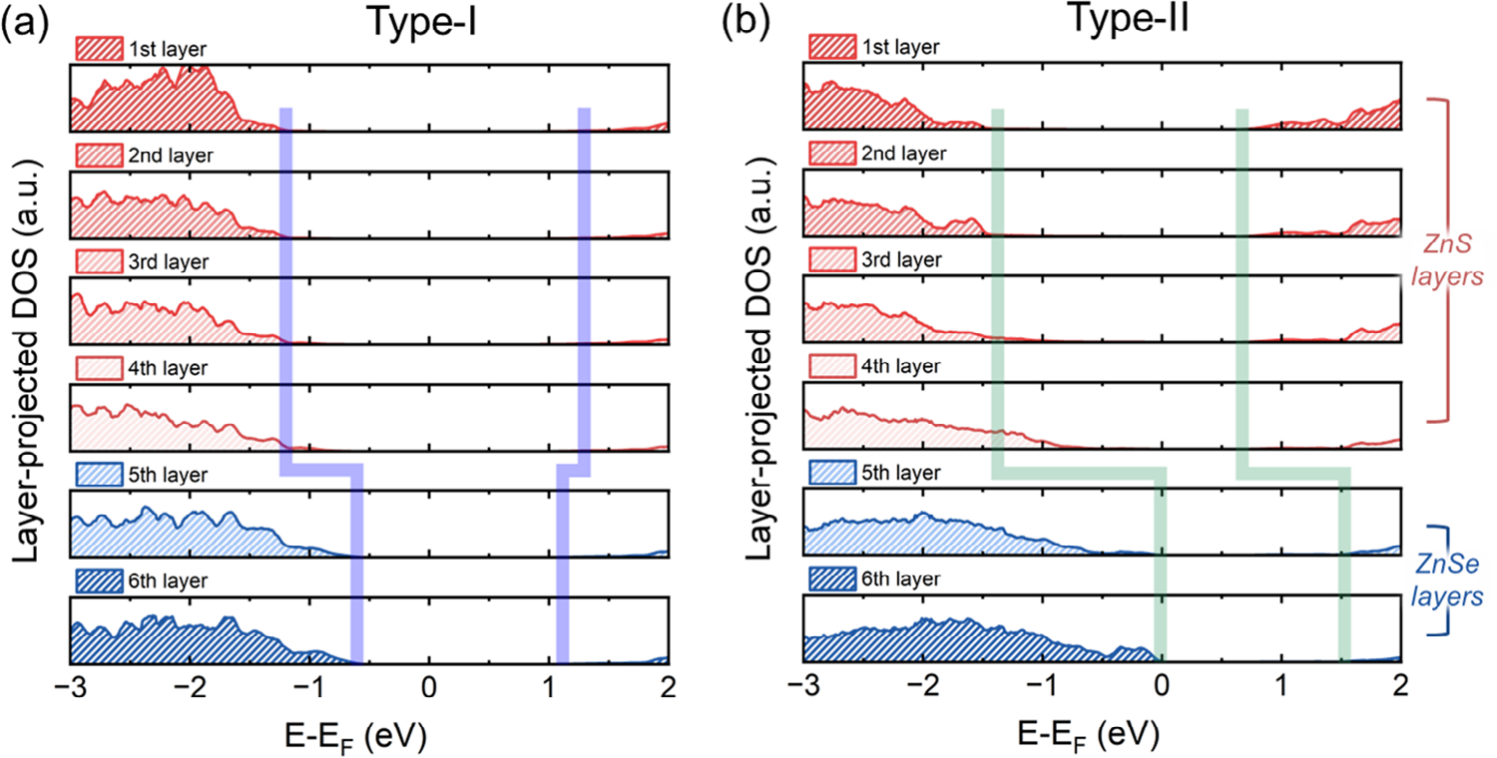
Layer‐resolved electronic structure of a ZnS/ZnSe heterostructure from DFT calculations. a) Layer‐resolved density of states (DOS) for the pristine structure. Blue lines indicate the VBM and CBM of each layer, revealing the intrinsic band alignment across the heterostructure. b) Layer‐projected DOS with vacancies, showing band edge modulation induced by defect propagation. Dashed blue and solid red lines denote the VBM and CBM of the pristine and defective structures, respectively.

The sequential formation of vacancies induces significant changes in the electronic structure of the ZnS/ZnSe heterostructure as shown in **Figure** **4**. As depicted in Figure 4a, the layer‐resolved DOS highlights the evolution of electronic properties from the ZnS surface through to the ZnSe interface. In the pristine structure, the ZnS outer layer exhibits a band gap of approximately 2.3 eV, which is wider than the 1.7 eV band gap of the ZnSe core layer. This configuration results in a type‐I band alignment: both the VBM and the CBM of ZnSe (highlighted by purple lines in Figure 4a) lie within the band gap of ZnS. Such an alignment confines both electrons and holes within the core region, supporting efficient photoluminescence. These results are consistent with prior DFT calculations and experimental data.^[^ ^41^, ^42^ ^]^

Figure 4b presents the layer‐resolved DOS in the presence of a sequential vacancy formation, labeled from layers 1 to 6. In this case, the Zn‐S vacancy pairs induce notable deviations in the band alignment compared to the pristine structure. Specifically, the VBM and CBM near the ZnS/ZnSe interface are shifted upward, while the Zn‐S vacancy pair near the surface causes the VBM and CBM of ZnS to shift downward. This redistribution of electronic states transforms the band alignment from type‐I to type‐II. In the type‐II alignment, holes predominantly localize near the ZnSe core, while electrons are drawn toward the ZnS shell. In the vicinity of the Zn‐S vacancy pairs, the spatial separation of carriers diminishes electron‐hole wave‐function overlaps, thereby suppressing radiative recombination. Although such band alignment can benefit applications requiring efficient charge separation, such as photovoltaics, it is detrimental for optoelectronic devices like light‐emitting diodes and QD‐based displays, where radiative recombination and photoluminescence efficiency are critical.

## 3 Conclusion 

In summary, we systematically investigated vacancy formation and electronic structure changes in ZnS/ZnSe heterostructures, focusing on their roles in the photodegradation mechanisms of InP/ZnSe/ZnS QDs. By constructing a ZnS/ZnSe slab exposing (110) surface, we assessed the stability of intrinsic vacancies and observed the evolution of the DOS across different layers. Our findings revealed that V_S_ are most likely to form at the surface of the ZnS shell. During sequential vacancy formation, both V_S_ and V_Zn_ exhibit reduced formation energies as they propagate inward, driven by charge compensation effects and local stabilization. The layer‐resolved DOS analysis provided crucial insights into the band alignment transitions. In the pristine structure, a type‐I band alignment confines both electrons and holes within the ZnSe layer, supporting efficient photoluminescence. However, the introduction of a vacancy chain shifts the band alignment to type‐II, facilitating spatial charge separation but compromising optoelectronic performance in light‐emitting applications.

Time‐resolved ESR spectroscopy further underscored the detrimental impact of pre‐existing sulfur vacancies on QD photostability. QDs containing initial s‐V_S_ exhibited considerably higher defect densities and reduced photostability compared with those without, and established a direct correlation between vacancy presence and long‐term performance decline. In addition, the DFT and GIPAW calculations enabled the quantification of the g‐values for Zn, S, and O vacancies, yielding ESR spectra that closely matched the experimental observations. These simulations confirmed the pivotal role of s‐V_S_ in driving defect propagation and altering the electronic structure.

Our findings highlight the critical importance of controlling surface and interfacial vacancy dynamics for enhancing the photostability and performance of InP/ZnSe/ZnS QDs. By minimizing the initial s‐V_S_ concentrations and controlling vacancy propagation, future QD materials can achieve improved photostability and device efficiency. Strategies such as defect passivation during synthesis, optimized shell thickness, and tailored surface treatments can reduce the adverse effects of vacancies. These insights pave the way for developing next‐generation QD‐based devices with enhanced resilience, efficiency, and durability and support advancements in optoelectronic technologies, such as quantum dot displays, photovoltaics, and photodetectors.

## 4 Experimental and Computational Methods

### Experimental Methods

The InP/ZnSe/ZnS QDs used in this work were synthesized at Samsung Display Co., Ltd., following the procedure reported by Baek et al.^[^ ^43^ ^]^ InP cores were prepared from indium acetate (In(OAc)_3_) and tris(trimethylsilyl)phosphine (TMS_3_P) in octadecene with palmitic acid ligands. The indium precursor and ligand were dissolved in octadecene, degassed under vacuum, and heated to ≈280 °C under nitrogen. At this temperature, a TMS_3_P solution in trioctylphosphine (TOP) was rapidly injected to initiate core formation. The resulting crude dispersion was purified and reserved for subsequent shell growth. For shelling, zinc oleate, zinc acetate, and oleic acid were dissolved in trioctylamine (TOA), degassed, and heated to ≈280 °C under N_2_. The temperature was then lowered to ≈180 °C to introduce the InP cores. After core addition, the mixture was reheated to ≈340 °C, at which point Zn‐oleate and Se/TOP were supplied to deposit the ZnSe shell. Finally, additional Zn‐oleate together with S/TOP was introduced to form the ZnS outer shell. The QD dispersions were stored in an inert glove box (O_2_ < 0.5 ppm) to minimize exposure to oxygen.

ESR measurements were performed with a Bruker E500 continuous‐wave X‐band spectrometer driven in absorption mode with a microwave frequency of v = 9.4 GHz. A helium gas flow cryostat was used at an operating temperature of 10 K. InP/ZnSe/ZnS QD powder samples were placed in a quartz sample tube and held in a microwave cavity. Photoexcited ESR measurements were also performed using a white light source coupled to the spectrometer resonator and operating at a power of 100 W mercury lamp. The wavelength of light was selected with a 450 nm bandpass filter. The ESR signal represents point defects such as S vacancies, Se vacancies, metal vacancies, oxidations (ZnO), and interstitial ions in InP/ZnSe/ZnS QD, and can identify the type of defect by extracting g‐factors from the following equation:

(3) g=hν/βH

where *h*, *ν*, *β*, and *H* indicate the Planck constant (6.626 × 10^−34^ J s), frequency of applied microwave (≈9.4 GHz), Bohr magneton (9.274 × 10^−24^ J T^−1^), and applied magnetic field, respectively. The spin density was calculated using the SpinCount & Spinfit software.

### Theoretical Calculations

The initial relaxation of defective structures was performed using a hybrid functional approach, employing the Heyd‐Scuseria‐Ernzerhof (HSE) potential,^[^ ^44^ ^]^ where the exact exchange fraction, α, was set to 0.375 and the inverse screening length, µ, was chosen as 0.2 Å^−1^ to limit the long‐range component of the exact exchange. The relaxation calculations were carried out using the Vienna ab initio simulation package (VASP) code^[^ ^45^, ^46^, ^47^ ^]^ using a well‐converged plane‐wave cutoff energy (500 eV) within the framework of the projector augmented wave (PAW) method.^[^ ^48^ ^]^ The supercells containing 128 atoms were used for bulk calculations, while slab models comprised 4 layers of ZnS at each surface and 7 layers of ZnSe, totaling 272 atoms.

For *g*‐tensor calculations, the Gauge Including Projector Augmented Waves (GIPAW) code was employed. While GIPAW does not yet support hybrid functionals, it incorporates Hubbard‐*U* corrections and is integrated with the QUANTUM ESPRESSO (QE) code.^[^ ^49^ ^]^ The procedure involves first determining a self‐consistent potential using a standard QE run. Subsequently, the GIPAW code calculates the first‐order induced current through density functional perturbation theory (DFPT) and uses this to extract various g‐tensor contributions, including the magnetic field generated by the first‐order current from the Biot‐Savart law, which leads to spin‐other‐orbit contributions. Moreover, the GIPAW code includes other relativistic corrections besides spin‐orbit coupling and distinguishes paramagnetic and diamagnetic contributions, as detailed in the referenced literature.^[^ ^50^, ^51^ ^]^ Subsequent ESR spectroscopy simulations were performed using the EasySpin software package.^[^ ^52^ ^]^

## Conflict of Interest

The authors declare no conflict of interest.

## Supporting information



Supporting Information

## Data Availability

The data that support the findings of this study are available from the corresponding author upon reasonable request.
