# Supplementary material for: Defect‐Cascades‐Induced Photodegradation in InP/ZnSe/ZnS Quantum Dots
Source: Adv Sci (Weinh). 2025 Nov 3;13(4):e15691. doi: 10.1002/advs.202515691 (PMC12822439; doi:10.1002/advs.202515691)
Supplement: Supplementary file 1 — Supporting Information [file ADVS-13-e15691-s001.docx]

**Defect-Cascades-Induced Photodegradation in InP/ZnSe/ZnS Quantum Dots**

*Yeongrok Jin^1,2^, Seongmun Kim^1^, Yeo-Geon Yoon^3^, Taekjoon Lee^3^, Jae Bok Chang^3^, Kyung Sig Lee^3^, Nari Ahn^3^, Ran Kim*^3^, Young-gil Park*^3^, and Jaekwang Lee^*1^*


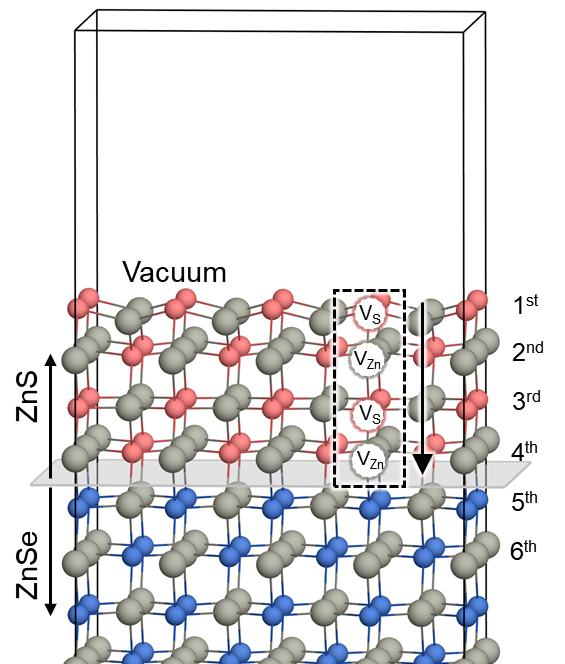


Figure S1 | Schematic representation of the slab structure for DFT calculations, featuring ZnSe at the center (layers 5–6) and ZnS on the surface (layers 1–4), oriented along the [110] direction. Layers are sequentially labeled from the vacuum-exposed surface (layer 1) to the ZnSe bulk (layer 6). A red box highlights sequential vacancy propagation, beginning with sulfur (S) and zinc (Zn) vacancies at the surface and continuing toward the interface region.

**Slab Geometry.** The slab models (272 atoms) comprised 15 atomic layers, exposing the (110) surfaces: four ZnS layers at each surface (layers 1–4 and 12–15) and seven ZnSe layers in the interior (layers 5–11). A 16 Å vacuum region was added along the surface normal to suppress spurious interactions. The in-plane lattice parameter was set to the arithmetic mean of the relaxed bulk ZnS and ZnSe values. During structural optimization, the lattice vectors were fixed while all atomic positions were fully relaxed.


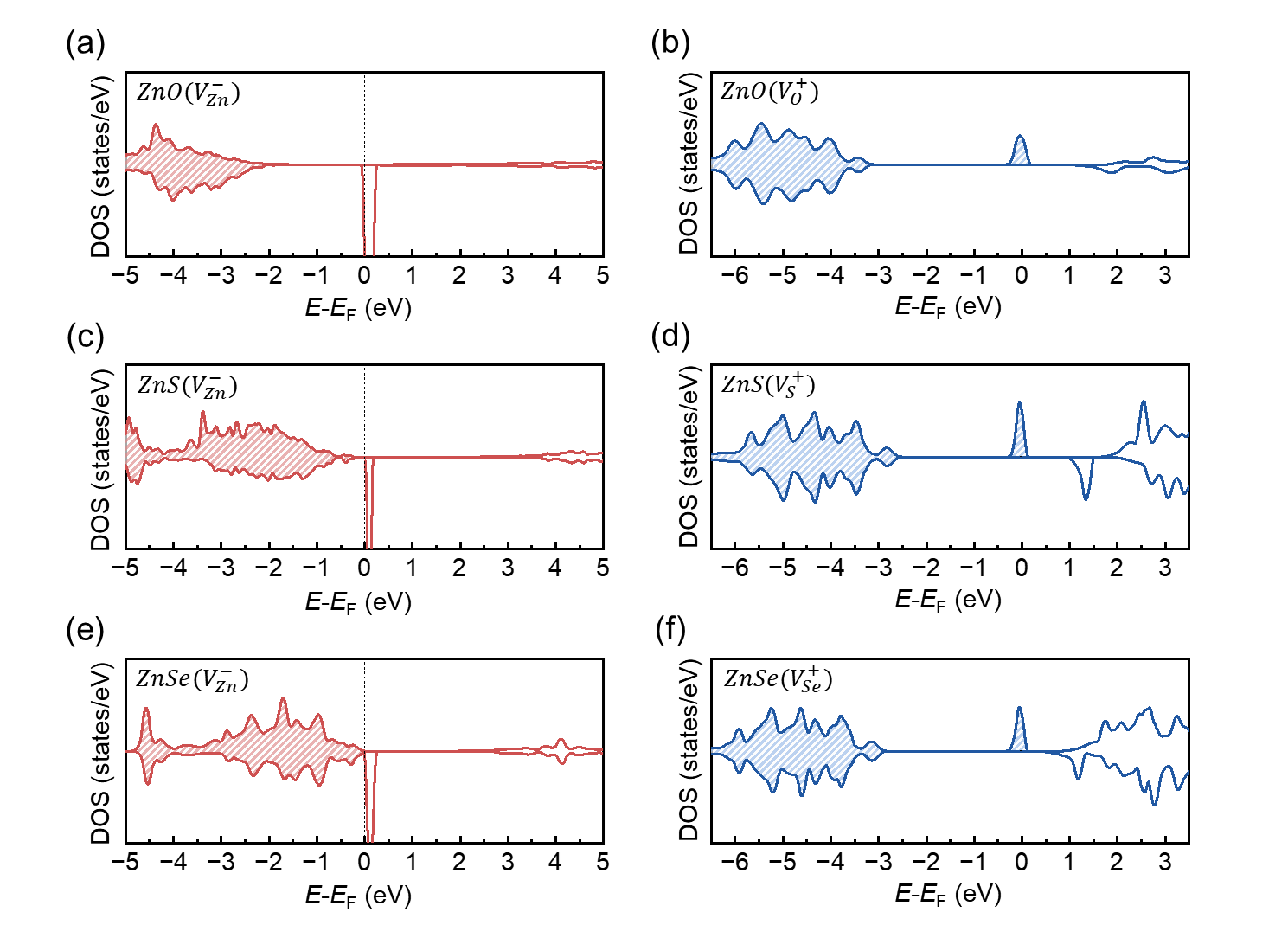


Figure S2 | The electronic structure of ZnX(X=O, S, Se). (a, c, e) Projected density of states (pDOS) for the p-orbital of X atom near Zn vacancy. (b, d, f) pDOS for the p-orbitals of the Zn atom near X vacancy.

**Density of States of Zn*X* (*X*=O, S, Se) with vacancies.** We performed density functional theory (DFT) calculations to gain deeper insight into the electronic properties of vacancies in Zn*X* (*X*=O, S, Se). Two types of defects were considered: singly negatively charged Zn vacancies (V_Zn_^-^) and positively charged X vacancies (V_X_^+^). Figure S2 illustrates the projected density of states (pDOS) of atoms neighboring the vacancies.

Our analysis reveals distinct electronic characteristics for each vacancy type. V_Zn_^-^ induces a localized hole state, as evident from the sharp empty state just above the Fermi energy. In contrast, V_X_^+^ introduces an additional electron into the system, resulting in occupied states just below the Fermi level. Notably, for Zn vacancies, the defect states in ZnO are closer to the conduction band compared to those in ZnS and ZnSe, reflecting material-specific differences in the band structure.

Another key observation is the nature of the conduction states associated with V_O_^+^, V_S_^+^, and V_Se_^+^. The conduction states for V_O_^+^ are broader and more dispersive than those for V_S_^+^ and V_Se_^+^, indicating stronger delocalization of electrons near oxygen vacancies. These characteristic highlights the distinct electronic behavior of defects in ZnO compared to ZnS and ZnSe, which could have significant implications for their electronic and optical properties.

**
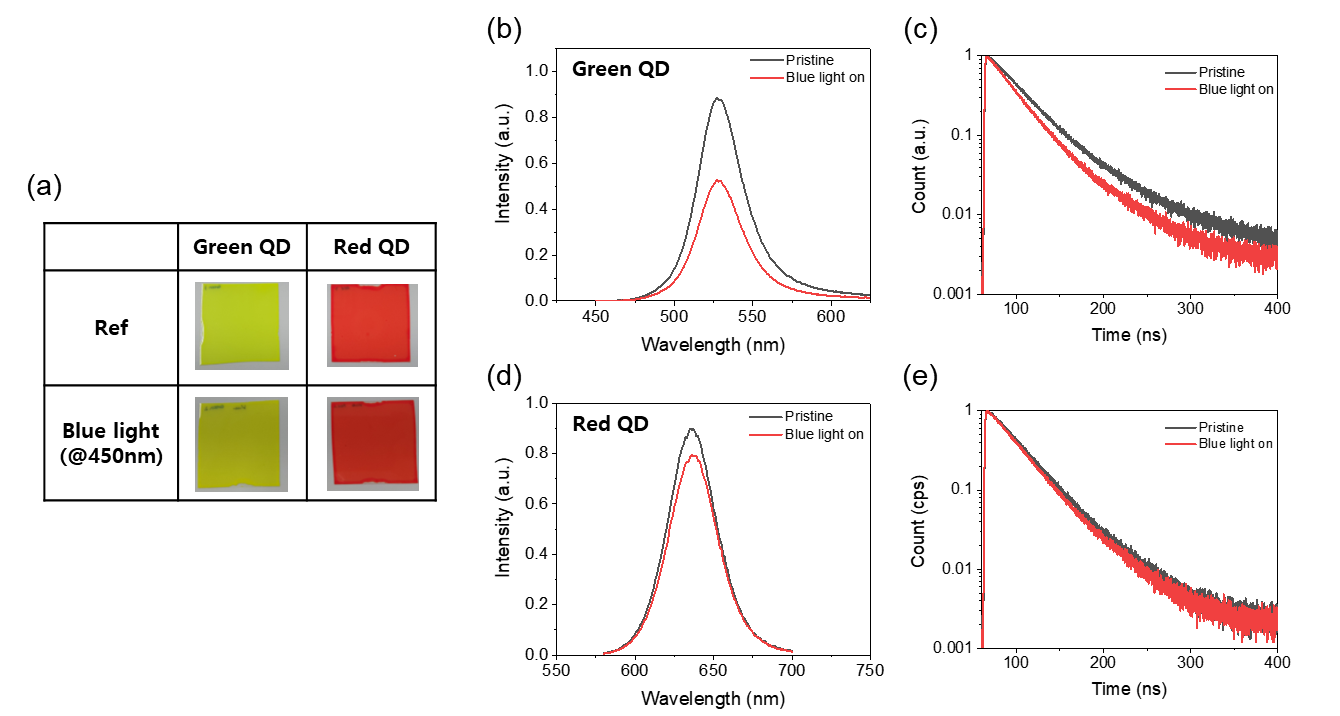
**

Figure S3 | Photoluminescence (PL) and time-resolved photoluminescence (TRPL) characterization of InP/ZnSe/ZnS QDs. (a) Representative photographs of QD films. (b) Photoluminescence (PL) spectra collected at room temperature show narrow-band emission; peak wavelength is summarized in Table S1. (c) Time-resolved PL (TRPL) decays recorded at the PL maxima; lifetimes are reported in Table S1.

**Photoluminescence (PL) and Time-Resolved PL (TRPL) Characterization.** Untreated InP/ZnSe/ZnS QD films were prepared and handled under inert atmosphere. “Blue-light exposure” refers to continuous illumination in the blue range (*λ* ≈ 450 nm) for 8 h at constant irradiance. Aliquots taken before (“untreated”) and after exposure were characterized under identical optical conditions. Room-temperature PL spectra were recorded from the films using above-band continuous-wave excitation. All spectra were background-subtracted and normalized to the acquisition time. Peak emission wavelength (*λ*_em_​) and full width at half maximum (FWHM) were obtained by single-peak least-squares fits (Gaussian model unless otherwise noted). For a Gaussian peak with standard deviation σ, FWHM was computed as $2\sqrt{2\ln2}\sigma$. TRPL decays were collected at the PL maxima using a pulsed source and time-correlated single-photon counting (or equivalent) detection. Steady-state PL shows narrow-band emission for both green and red QDs (Figure S3(b)). In the untreated state, *λ*_em_​ is 528/635 nm for green/red QDs, shifting marginally to 529/636 nm after 8 h of blue-light exposure (Table S1). This slight red-shift is consistent with subtle surface or field effects without major changes in size distribution. FWHM values remain within experimental variation, indicating that spectral broadening due to size dispersion is minimal following exposure. By contrast, TRPL reveals a clearer change (Figure S3(c)). The amplitude-weighted average lifetime decreases from 40.5/36.4 ns (green/red, untreated) to 33.7/34.8 ns after exposure (Table S1). In terms of the total decay rate *Γ*=1/⟨*τ*⟩, this corresponds to an increase of ~20% (green) and ~4.6% (red). These optical trends establish a pristine baseline and demonstrate that blue-light aging primarily affects recombination dynamics (lifetime) rather than size distribution (FWHM), providing a sensitive readout of emerging nonradiative processes.

**Table S1 |** PL peak emission wavelength (*λ*_em_​) and average PL lifetime (ns) of green- and red-emitting InP/ZnSe/ZnS QDs before (untreated) and after 8 h of blue-light exposure. A slight redshift in λ_em_ and a reduction in PL lifetime are observed after exposure.

| **Sample** | ***λ*_em_ (nm)** | | **FWHM (nm)** | | **Lifetime (ns)** | |
| --- | --- | --- | --- | --- | --- | --- |
|  | **Green** | **Red** | **Green** | **Red** | **Green** | **Red** |
| **Untreated** | **528** | **635** | **35** | **37** | **40.5** | **36.4** |
| **Blue 8hr** | **529** | **636** | **33** | **36** | **33.7** | **34.8** |


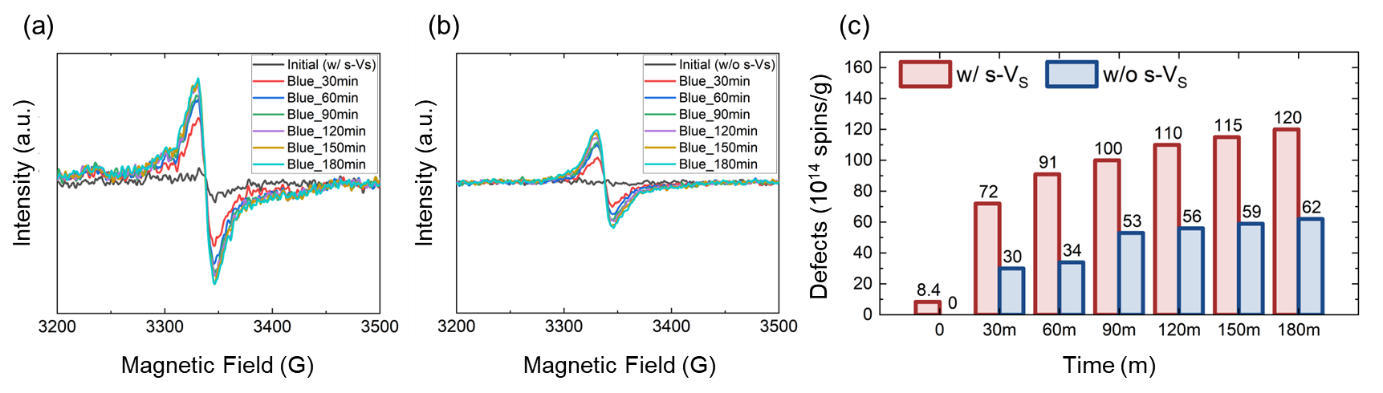


Figure S4 | ESR experimental results for InP/ZnSe/ZnS QDs w/ and w/o initial s-Vs. (a) and (b) show the corresponding ESR spectra for each case, with representing the spectra when (a) initial S vacancy is present and (b) when no initial S vacancy is present. The ESR spectra were recorded at intervals of 0, 30, 60, 90, 120, 150 and 180 minutes. (c) Quantification of defects in QDs as detected in ESR spectroscopy.

**Extended ESR experimental results for InP/ZnSe/ZnS QDs.** We carried out additional in-situ ESR measurements extending the time window to 180 minutes (Figure S4). Spectra were acquired every 30 minutes from 0 to 180 minutes. Because we did not average multiple runs at each time point; accordingly, the data are noisier than in Figure 1, but the underlying trend is clear. Figures S4(a,b) display representative spectra, and Figure S4(c) summarizes spin-counted defect densities (spins g^-1^). At 30 minutes, the defect-density difference between QDs with initial s-V_S_ and those without is 4.2$\times$10^15^ spins g^-1^; by 180 minutes this gap increases to 5.8$\times$10^15^ spins g^-1^. Both cases show a rapid rise during the first -30 minutes followed by an approach to saturation within ~2–3 hours (consistent with Figures 2(d,e)), but the widening defect-population gap with time supports the larger EQE divergence observed in device-level aging over hundreds of hours.
